# The Effect of Metabolic Syndrome and Its Individual Components on Renal Function: A Meta-Analysis

**DOI:** 10.3390/jcm12041614

**Published:** 2023-02-17

**Authors:** Xu Li, Qichen Liang, Junfeng Zhong, Liangying Gan, Li Zuo

**Affiliations:** Department of Nephrology, Peking University People’s Hospital, No. 11 Xizhimen South Street, Xicheng District, Beijing 100044, China

**Keywords:** chronic kidney disease, renal function, metabolic syndrome, meta-analysis

## Abstract

Background: Observational studies have reported inconsistent findings in the relationship between metabolic syndrome (MetS), its components, and loss of renal function, mainly including eGFR decline, new-onset CKD, and ESRD. This meta-analysis was performed to investigate their potential associations. Methods: PubMed and EMBASE were systematically searched from their inception to 21 July 2022. Observational cohort studies in English assessing the risk of renal dysfunction in individuals with MetS were identified. Risk estimates and their 95% confidence intervals (CIs) were extracted and pooled using the random-effects approach. Results: A total of 32 studies with 413,621 participants were included in the meta-analysis. MetS contributed to higher risks of renal dysfunction (RR = 1.50, 95% CI = 1.39–1.61) and, specifically, rapid decline in eGFR (RR 1.31, 95% CI 1.13–1.51), new-onset CKD (RR 1.47, 95% CI 1.37–1.58), as well as ESRD (RR 1.55, 95% CI 1.08–2.22). Moreover, all individual components of MetS were significantly associated with renal dysfunction, while elevated BP conveyed the highest risk (RR = 1.37, 95% CI = 1.29–1.46), impaired fasting glucose with the lowest and diabetic-dependent risk (RR = 1.20, 95% CI = 1.09–1.33). Conclusions: Individuals with MetS and its components are at higher risk of renal dysfunction.

## 1. Introduction

Chronic kidney disease (CKD) is an emerging major global public health problem, with increasing incidence and prevalence, adverse outcomes, and substantial cost [1]. Besides its progression to end-stage renal disease (ESRD), CKD is an independent risk factor for cardiovascular disease, cognitive dysfunction, hospitalization, and all-cause mortality [2]. Therefore, identification and treatment of modifiable risk factors to prevent or slow the decline in kidney function are receiving increasing attention.

Metabolic syndrome (MetS) is a cluster of metabolic traits including obesity, impaired fasting glucose (IFG), elevated blood pressure (BP), and dyslipidemia [3]. Its independent link with CKD development and progression has also been increasingly discussed but with inconsistent findings. A population-based study revealed that the impact of MetS on CKD progression is significant only in non-diabetic early-stage CKD (stages 1–3), but not non-diabetic late-stage and diabetic CKD patients [4]. Another study reported a strong association between MetS components and CKD, independent of diabetes grades [5]. Furthermore, a cohort study reported no association of MetS with incident CKD despite a positive and significant risk of estimated glomerular filtration rate (eGFR) decline [6]. Several meta-analyses were performed to resolve these controversies. A previous meta-analysis of 11 cohort studies suggested that MetS could foster development of CKD [7]. Another meta-analysis of 57 studies showed that MetS and its components are potential risk factors for albuminuria and proteinuria [8]. However, prior meta-analysis only included studies on subjects free of CKD. No meta-analyses were conducted to investigate the relation of MetS and its components to renal events including eGFR decline or progression to ESRD. Additionally, there are also some discrepancies regarding the role of each single component of MetS on renal function.

Given the clinical uncertainty, we conducted this meta-analysis to evaluate the effect of MetS on renal function, giving particular attention to the risk conveyed by each trait of MetS and updating the evidence in the context of individuals with reduced baseline renal function and a broader kidney outcome.

## 2. Materials and Methods

This meta-analysis was registered in the International Prospective Register of Systematic Reviews—PROSPERO (CRD42022349368) and conducted in accordance with the Preferred Reporting Items for Systematic Reviews and Meta-analyses (PRISMA) guidelines [9,10].

### 2.1. Search Strategy

A comprehensive literature search was performed to identify related studies in PubMed and Embase databases from their inception to 21 July 2022. Medical subject headings (MeSH) terms and synonyms for the different terms including “metabolic syndrome”, “kidney function”, “chronic kidney disease” and “end-stage renal disease” were used and combined with Boolean operators and wildcards (Supplement S1). A manual search for additional relevant studies using references from retrieved articles was also performed.

### 2.2. Study Selection

Studies were included in the current meta-analysis if they fulfilled the following criteria: (1) observational cohort studies; (2) studies with a clear definition of MetS. The risk estimate in individuals with three or more prespecified metabolic abnormalities was considered, in which studies did not report the definition; (3) studies reporting following outcome measures: rapid eGFR decline, 50% reduction in eGFR or doubling of serum creatinine, new-onset CKD, progression to ESRD, or a composite of the above components. The definitions of each outcome were varied across studies, and we used these outcomes reported in each study; (4) relative risks (RRs) [or odds ratios (ORs) or hazard ratios (HRs)] with 95% confidence intervals (CIs) could be obtained; (5) articles published in English; (6) full text available.

Studies were excluded if they met the following criteria: (1) non-original articles (reviews, editorials, or letter to the editor); (2) studies with overlapped subjects; (3) studies that reported risk estimates for metabolic syndrome components only; (4) studies that reported effect estimates only by subgroups instead of the total sample; (5) studies reporting a composite outcome including death. Two reviewers (X.L., Q.C.L.) independently screened studies against the inclusion and exclusion criteria. A third author (J.F.Z.) adjudicated any discordance in assessments.

### 2.3. Data Extraction

After determining the qualified articles, the following data were extracted from the included studies: name of the first author, year of publication, study location, participant characteristics including sample size, gender proportion, mean age, follow-up duration, MetS definition, number of renal events, definition used for kidney outcome, risk estimates and their 95% Cis, and variables adjusted for multivariate analysis. We preferentially adopted outcomes using the modified NCEP-ATP III criteria for Asian participants for this analysis, but we accepted data on other definitions where these were the only ones reported. If one study had reported more than one kidney outcome, we extracted the data for the composite kidney outcome for the primary risk estimates as well as the data for each kidney outcome component, when available. If the study didn’t report the overall effect, the data related to declined GFR, a valid end point for chronic kidney damage [11,12], was selected to pool the primary risk estimate. When one study presented different adjustment variables, we collected data for the most adjusted model. In order to separate the influence of each single component of MetS, we also collected risk estimates for each single trait. To improve accuracy and critical appraisal, data extraction was performed by two independent reviewers (X.L., Q.C.L.). Any discrepancies were resolved in consultation with a third author (J.F.Z.).

### 2.4. Quality Assessment

The quality of studies was assessed according to the Newcastlee-Ottawa Scale (NOS) for quality assessment of cohort studies [13] by two of the authors independently (X.L., Q.C.L.), and any discrepancies were resolved via discussion or referral to a third reviewer (J.F.Z.). A final score 7–9 was classified as high quality.

### 2.5. Statistical Analysis

In order to examine the effect of MetS and its components on renal function, the study-specific maximally adjusted RRs, ORs, or HRs were collected. The primary outcome was defined as renal dysfunction, a composite of rapid eGFR decline, new-onset CKD, 50% reduction in eGFR, or doubling of serum creatinine and ESRD. We also estimated the risk effect on each kidney outcome, when available. HRs and ORs were converted to RRs before pooling based on the formulae in Supplement S2 [14]. Heterogeneity was quantitatively assessed using I^2^ statistic [15] with 25%, 50%, and 75% considered moderate, substantial, and considerable heterogeneity, respectively. Random-effect models were applied allowing for between-study variability by weighting studies using a combination of intra- and inter-study variance [16].

We compared the effect of MetS on renal function in the following subgroups: age range of participants, country, follow-up duration, diabetes status, and baseline kidney function. Leave-one-out sensitivity analyses was used to identify the stability of the results. Publication bias was assessed using the Egger’s test, and funnel plots were drawn [17]. The statistical significance level was set at *p* value < 0.05. Analyses were performed using Comprehensive Meta-Analysis (Englewood, NJ, USA), version 3.0.

## 3. Results

### 3.1. Study Characteristics

**Figure 1:**
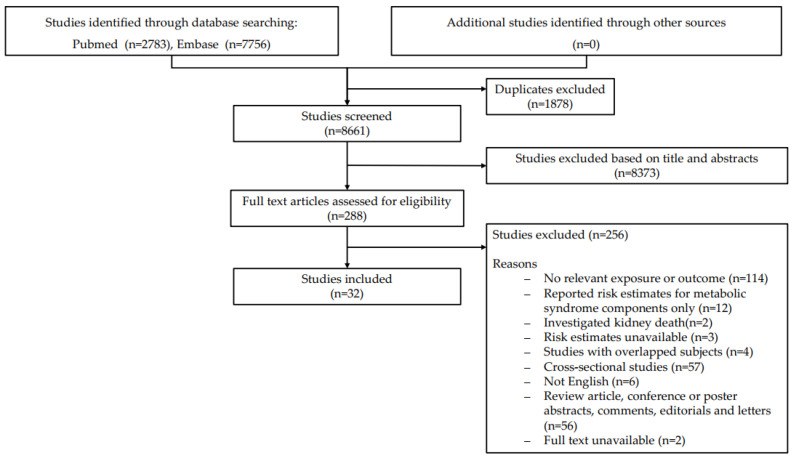
PRISMA Diagram depicting the screening and study selection process.

The database searches identified 10,539 papers. After removal of duplicates, the included articles were selected from a pool of 8661 articles obtained from digital sources and a manual search. Of these, 288 papers retrieved for full-text screening against the inclusion and exclusion criteria. Eventually, a total of 32 studies published between 2005 and 2022 were included (Figure 1).

The study participants were followed up for an average of nearly five years. Mean patient age ranged from 37 [18] to 73 [19] years, and the proportion of males ranged between 26% [20] and 100% [18]. Of the 32 studies, most were conducted on the general population, while five studies [20,21,22,23,24] were conducted on diabetic patients, eight studies [18,25,26,27,28,29,30,31] on non-diabetic individuals. Eleven studies [5,19,20,28,31,32,33,34,35,36] were performed in middle-aged or elderly participants (≥40 years). Moreover, seven studies [19,22,23,24,37,38,39] enrolled subjects with reduced baseline eGFR, while the other 25 studies [5,6,18,20,21,25,26,27,28,29,30,31,32,33,34,35,36,40,41,42,43,44,45,46,47] excluded CKD patients. Further study-level detail regarding the characteristics of included studies is summarized within the Supplement S3.

Quality assessments of all included studies showed a Newcastle-Ottawa score range of 7–9 (out of 9), which indicates a good quality (Supplement S4).

### 3.2. Overall Analysis of Pooled Data

**Figure 2:**
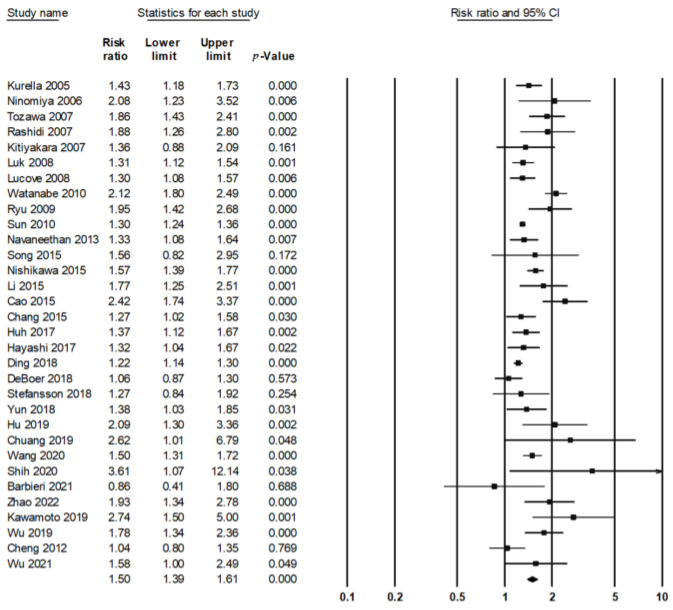
Forest plot showing overall analysis on the effect of metabolic syndrome on renal function [5,6,18,19,20,21,22,23,24,25,26,27,28,29,30,31,32,33,34,35,36,37,38,39,40,41,42,43,44,45,46,47].

The association between MetS and renal dysfunction was investigated in 32 studies, involving 413,621 participants of which 93,944 subjects had MetS, and 34,488 renal events were reported. The presence of metabolic syndrome increased the risk of renal dysfunction by 50% (RR = 1.50, 95% CI = 1.39–1.61), with evidence of moderate heterogeneity across studies (I^2^ = 72.57%, *p* < 0.001) (Figure 2, Table 1). After excluding one study at a time, the sensitivity analysis confirmed the increased risk of MetS for renal dysfunction [RR with 95%CI ranging from 1.47 (1.37–1.58) to 1.52 (1.40–1.65)] (Supplement S5). Specifically, MetS was associated with increases in the risks of rapid decline in eGFR (RR 1.31, 95% CI 1.13–1.51, I^2^ = 64.16%, *p* = 0.007), new-onset CKD (RR 1.47, 95% CI 1.37–1.58, I^2^ = 61.69%, *p* = 0.001), as well as ESRD (RR 1.55, 95% CI 1.08–2.22, I^2^ = 66.56%, *p* = 0.084) (Table 2).

In subgroup analysis, the pooled risk based on studies only enrolling mediate-aged or elderly (≥40 years) participants was equal to the original estimate (RR 1.51, 95% CI 1.33–1.71, I^2^ = 51.92%, *p* = 0.023). There were no differences among follow-up duration subgroups. Additionally, the strength and direction of this association was found to be independent of diabetes status; however, diabetic patients with MetS did confer a higher risk than non-diabetic subjects (pooled RR 1.61 vs. 1.41, *p* for heterogeneity = 0.237). Similarly, the risk estimates did not differ among participants with or without baseline renal dysfunction (pooled RR 1.52 vs. 1.38; *p* for heterogeneity = 0.357) (Table 1).

### 3.3. The Effect of MetS Components and Renal Function

The pooled risk estimates of renal dysfunction for individual MetS components are presented in Table 3. Consistent with the results for MetS, pooled RRs (95% CI) were 1.27 (1.19–1.37) for obesity, 1.20 (1.09–1.33) for IFG, 1.42 (1.27–1.59) for elevated BP, 1.25 (1.15–1.36) for increased triglycerides (TG), and 1.24 (1.15–1.34) for reduced high-density lipoprotein-cholesterol (HDL-C). This association was supported among all components in analysis stratified by age range of participants, and follow-up duration. A stronger association between elevated BP and renal dysfunction was observed in non-Asian populations (RR 2.25, 95% CI 1.70–2.98) compared with Asian populations (RR 1.33, 95% CI 1.20–1.48). Additionally, the relationship between IFG and renal dysfunction was a diabetes-dependent association, where non-diabetic subjects conferred a non-significant increased risk (RR 1.01, 95% CI 0.94–1.08). Regarding increased TG and reduced HDL-C, the association was not prominent in individuals with reduced baseline renal function (RR 1.15, 95% CI 0.94–1.40 for increased TG, RR 1.31, 95% CI 0.93–1.38 for reduced HDL-C, Supplement S6). In the sensitivity analysis, the associations between each of the components and the risk of renal dysfunction were not changed remarkably.

### 3.4. Evaluation of Publication Bias

After visually examining the funnel plots and performing Egger tests for every parameter, possible publication bias was observed for the associations of MetS (*t* = 3.27, *p* = 0.003, Table 1) and increased TG (*t* = 5.03, *p* = 0.010, Table 3, Supplement S7).

## 4. Discussion

Our meta-analysis of 32 cohort studies including 413,621 participants found that the presence of MetS predicted loss of renal function (RR = 1.50, 95% CI = 1.39–1.61). The risk estimate kept consistent regardless of different age range, duration of follow-up, country, diabetic status, or baseline kidney function. Besides CKD development, our findings revealed that MetS maintained its prognostic value for disease progression, including rapid eGFR decline and ESRD. The risk conveyed by all individual components of MetS varied and was strongest for elevated BP.

Prior meta-analysis studies have suggested that individuals with MetS are at increased risk of developing CKD, reflected by albuminuria or proteinuria [8] and eGFR decline [7,48]. The present study supports the results of previous studies, in which the presence of MetS and all five components were related to the onset of CKD, and reports a similar magnitude of the association. Besides incident CKD, several studies examined the role of MetS and its components in the risk of kidney disease progression, but concluded conflicting results. A large prospective cohort across the United States showed that individuals with MetS were found to have a 2-fold higher risk of developing ESRD as compared to the those without MetS [49], demonstrating the important role MetS plays in CKD progression. In contrast, the secondary analysis of the African-American Study revealed that the association of MetS and CKD acceleration is confounded by other factors, and MetS is not independently related to CKD progression [50]. No previous meta-analysis attempted to evaluate the association between MetS and progression of established CKD. Our findings extend those of previous studies. In this analysis, a broader composite kidney outcome was examined, including CKD development, doubling of serum creatinine or eGFR reduction more than 50%, rapid eGFR decline, and progression to ESRD, among individuals with normal or reduced renal function. The present study supported a significant association between MetS and renal dysfunction acceleration, suggesting that apart from incident CKD, MetS could also be an independent predictor for disease progression. 

The meta-analysis of individual components was done to explore the relative contribution of the individual components of MetS to renal dysfunction risk. Diabetes and hypertension are the leading causes of both CKD and ESRD in all developed and many developing countries [51]. However, the risk, in this study, was found to be strongest for elevated BP, but weakest for IFG. The association between elevated BP with renal insufficiency was confirmed by a few studies [52,53]. The magnitude of the risk in this study is in line with previous meta-analyses [48] which supported a conclusion of positive association between elevated BP and adverse kidney function with a RR of 1.37 (1.29–1.46). A stronger association between BP elevations and renal dysfunction was reported in non-Asian populations compared with Asian populations in the subgroup analysis. However, according to previous research, the presence of elevated BP is associated with greater albuminuria and proteinuria risk regardless of nationality [8]. No evidence of regional differences was observed regarding the impact of prehypertension on new-onset CKD [54] and progression to ESRD [55]. Such a discrepancy might be driven by one of the three studies included in the non-Asian subgroup reporting a remarkable influence of hypertension [30]. Meanwhile, baseline characteristics of individuals in this study showed a higher prevalence of elevated BP with insufficient treatment and control [30]. On the other hand, previous studies investigating the association between glycemic status and kidney disease have contradictory conclusions. A recent meta-analysis suggested that prediabetes, including IFG, impaired glucose tolerance (IGT), or elevated glycated hemoglobin A1c, modestly increased the risk of CKD (RR = 1.11, 95% CI 1.02–1.21) [56]. A Mendelian randomization study found that IFG was not causally associated with CKD development in non-diabetic population [57]. The present study demonstrated that people with baseline IFG were at a significantly but modestly increased risk of renal dysfunction, in agreement with the early study [7]. Such a discrepancy might be attributable to the different characteristics of the study participants. Studies addressing IFG as part of MetS may have included people with diabetes at baseline or during follow-up and not included people with IGT. Therefore, the relationship between IFG and renal dysfunction may vary depending on other glycemic exposures. This meta-analysis was done to evaluate the effect among subjects with MetS. Hence, the risk estimates should be interpreted in the context of MetS. IFG is not commonly presented alone, but regarded as a result of complex interactions among components of MetS [58], especially in CKD, a population with a high frequency of hypertension, diabetes, altered lipid metabolism, and preexisting cardiovascular disease [59,60]. Therefore, the current study, with a large sample size and strong statistical power, was believed to lead to an accurate estimation of the risk related to IFG. 

This study also revealed that apart from hyperglycemia and raised BP, as traditional risk factors of CKD, other components of MetS, including obesity, increased triglyceride, and reduced HDL-cholesterol are also reported to be important factors in new-onset CKD and subsequent disease progression in observational studies [61,62,63] and meta-analyses [48,64,65]. Targeting disturbances of renal energy metabolism is a promising approach to addressing the current epidemic of metabolic disease-induced renal diseases. Although studies clearly indicate that obesity-associated CKD can be driven by diabetes and hypertension [66], a vast number of researches have established obesity as an independent risk factor for promoting new-onset or accelerating pre-existing CKD [67,68]. In an individual-level meta-analysis of 5.5 million adults in 39 general population cohorts, BMI levels of 30, 35, and 40 kg/m^2^ were associated with 18%, 69%, and 102% higher risk of eGFR decline ≥40%, and the association was fairly similar in patients with and without baseline CKD [69]. Mechanistically, inflammation, insulin resistance, renal hemodynamic changes, and lipid metabolism disorders are all involved in the development and progression of obesity-induced nephropathy [66]. Dyslipidemia is prevalent among CKD patients. Increased TG levels, decreased and dysfunctional HDL-C, and varying levels of low-density lipoprotein-cholesterol contribute to the dyslipidemic profile in CKD populations [70]. The association of dyslipidemia with renal dysfunction has been examined across stages of CKD. A cohort study identified the elevated TG as a risk factor driving the observed association between MetS and renal function decline among patients without CKD [26]. Data from another study demonstrated that high TG levels were associated with a higher incidence of CKD and a faster renal function decline in non-CKD and CKD stage 3, yet showed no or inverse associations with time to ESRD in CKD stages 4–5 [71]. There was also no significant correlation reported between TG levels and HDL-C levels with progression to renal replacement therapy and rapid renal progression in CKD stages 3–5 in another study [72]. HDL-C was identified in previous research to play a crucial role in the development of CKD [73]. Another cohort study enrolling subjects with CKD stages 3–4 reported that the link between reduced HDL-C and progression to kidney failure was abolished after adjustment for relevant covariates in multivariate analyses [74]. In this study, the associations between disorders of either TG or HDL-C and renal impairment was only observed in baseline non-CKD subgroup. A possible explanation is that in advanced CKD, especially ESRD patients, the effects of hyperlipidemia are masked in the presence of other powerful traditional CVD risk factors. Altered energy metabolism, inflammation, malnutrition, and protein energy wasting may play a more prominent role in disease progression [71]. In our findings, the proven association between dyslipidemia and renal dysfunction in individuals in the non-CKD subgroup, rather than the baseline CKD subgroup, enables nephrologists to focus on the early stages of CKD, which may lead to the earlier application of interventions for metabolic abnormalities, such as lifestyle modifications or even pharmaceutical treatments. Besides, previous studies also identified that fat distribution has a critical role, as abdominal, not general obesity, on adverse renal outcomes [75]. However, subgroup analysis by fat distribution was not conducted in the present study because some studies substituted one with the other in part of their participants when the data for those individuals was lacking.

Large pooled sample size, detailed subgroup analysis, and a prespecified study protocol that included a systematic search of PubMed and EMBASE databases for cohort studies were the chief strengths of the present study. However, several limitations should be recognized. First, we noted significant clinical heterogeneity in the analysis which limited the interpretation of our findings. The heterogeneity for renal dysfunction in relation to the presence of MetS could not be explained on the basis of age range, geographic region, duration of follow-up, diabetic status, or baseline renal function. Other underlying diseases, and variables used among studies, could have contributed to the heterogeneity. In addition, we focused on the baseline metabolic status, while the duration of MetS or the metabolic status during follow-up might impact the result. Secondly, the present meta-analysis has focused only on papers published in English. Those reported in other languages may be the source of publication bias. Thirdly, the inclusion of proteinuria or albuminuria may strengthen the findings as extra confirmation of renal dysfunction. However, another recent meta-analysis with 10,603,067 subjects from 57 studies on this topic had been performed [8], so it was not included in this study. Also, studies investigating kidney outcomes including renal death were not meta-analyzed and the association should be investigated further in future studies. Finally, the results for each component of kidney outcomes of individual traits of MetS were not pooled because of the small number of studies. The association between each component and renal dysfuntion has been broadly discussed within or beyond the context of MetS. The risk conveyed by some particular components for the incidence of CKD [64,76,77] or ESRD [55] has been reported in previous meta-analyses.

## 5. Conclusions

The present meta-analysis showed that there was a remarkable association between MetS, its components, and the risk of renal dysfunction. Among individual components of the syndrome, the most predictive factor was elevated BP. Identifying risk contribution will help clinicians prioritize treatment for patients with MetS. These findings may have important clinical implications in terms of risk stratification and preventive strategies.

## Figures and Tables

**Table 1:** Main analyses and subgroup analyses for renal dysfunction in relation to the presence of metabolic syndrome.

Subgroup	Number of Studies/Participants	Test of Association	Test of Heterogeneity		Publication Bias	
		OR (95% CI)	I^2^ (%)	*p*	*t*	*p*
Overall	32/413,621	1.50 (1.39–1.61)	72.57	<0.001	3.27	0.003
Age range						
≥40 years	11/46,985	1.51 (1.33–1.71)	51.92	0.023	2.20	0.055
Median follow-up				0.713		
<5 years	18/318,137	1.48 (1.35–1.62)	67.95			
≥5 years	14/95,484	1.52 (1.36–1.69)	70.25			
Country				0.051		
Asian	25/366,576	1.56 (1.43–1.69)	76.55			
Non-Asian	7/47,045	1.30 (1.15–1.47)	36.08			
Diabetic status				0.237		
Diabetics	5/14,866	1.61 (1.32–1.96)	59.05			
Non-diabetics	8/218,044	1.41 (1.27–1.56)	67.28			
Baseline renal function				0.357		
Normal	25/382,295	1.52 (1.40–1.65)	75.91			
Reduced	7/31,326	1.38 (1.14–1.67)	54.86			

Renal dysfunction is defined as a composite of rapid eGFR decline, new-onset CKD, 50% reduction in eGFR, or doubling of serum creatinine and ESRD. I^2^ refers to homogeneity statistics within subgroups; *p* for heterogeneity refers to homogeneity statistics across subgroups. CI, confidence interval; OR, odd ratio.

**Table 2:** Effect of metabolic syndrome on kidney outcomes.

Kidney Outcome	Number of Studies/Participants	Test of Association	Test of Heterogeneity		Publication Bias	
		OR (95% CI)	I^2^ (%)	*p*	*t*	*p*
Rapid eGFR decline	8/25,403	1.31 (1.13–1.51)	64.16	0.007	1.78	0.126
New-onset CKD	22/341,955	1.47 (1.37–1.58)	61.69	<0.001	4.15	0.001
ESRD	3/26,445	1.55 (1.08–2.22)	66.56	0.084	0.14	0.910

CKD, chronic kidney disease; ESRD, end-stage renal disease; eGFR, estimated glomerular filtration rate.

**Table 3:** Main analyses for renal dysfunction in relation to components of metabolic syndrome.

Risk Factor	Number of Studies/Participants	Test of Association	Test of Heterogeneity		Publication Bias	
		OR (95% CI)	I^2^ (%)	*p*	*t*	*p*
IFG	18/362,546	1.20 (1.09–1.33)	85.82	<0.001	0.634	0.591
Elevated BP	20/362,875	1.42 (1.27–1.59)	87.42	<0.001	1.97	0.065
Obesity	19/339,572	1.27 (1.19–1.37)	68.78	<0.001	0.91	0.376
Increased TG	20/368,786	1.25 (1.15–1.36)	92.93	<0.001	5.03	0.010
Reduced HDL-C	18/337,089	1.24 (1.15–1.34)	87.19	<0.001	1.45	0.284

Renal dysfunction is defined as a composite of rapid eGFR decline, new-onset CKD, 50% reduction in eGFR, or doubling of serum creatinine and ESRD. I^2^ and *p* refer to homogeneity statistics within subgroups. BP, blood pressure; CI, confidence interval; HDL-C, high-density lipoprotein-cholesterol; IFG, impaired fasting glucose; OR, odd ratio; TG, triglycerides.

## Data Availability

Not applicable.

## Supplementary Materials

The following supporting information can be downloaded at: https://www.mdpi.com/article/10.3390/jcm12041614/s1, Supplement S1: Full search strategies; Supplement S2: Conversion of OR to RR; Supplement S3: Characteristics of the included studies; Supplement S4: Risk of bias of the included studies; Supplement S5: Sensitivity analysis; Supplement S6: Subgroup analyses for individual components of metabolic syndrome; Supplement S7: Funnel plot showing publication bias of studies on the association between metabolic syndrome and renal dysfunction. References [5,6,18,19,20,21,22,23,24,25,26,27,28,29,30,31,32,33,34,35,36,37] are cited in Supplementary Materials.
