# Supplementary material for: The Effect of Metabolic Syndrome and Its Individual Components on Renal Function: A Meta-Analysis
Source: J Clin Med. 2023 Feb 17;12(4):1614. doi: 10.3390/jcm12041614 (PMC9962508; doi:10.3390/jcm12041614)
Supplement: Supplementary file 1 [file jcm-12-01614-s001.zip › jcm-2139503-supplementary.pdf]

Supplementary Material

Supplement S1. Full search strategies

Summary of hits
10539 Identified
1878 Duplicates removed
288 Screened for full text review

PUBMED
(kidney[mh] OR Kidney Function Tests[mh] OR kidney diseases[mh] OR Renal Insufficiency, Chronic[mh] OR (albumins[mh] AND creatinine[mh]) OR cystatin C[mh] OR (kidney*[tiab] OR renal OR nephro*[tiab] OR glomeru*[tiab] OR ckd OR microalbuminuri*[tiab] OR (micro ADJ albuminuri*[tiab]) OR (albumin*[tiab] AND creatinin*[tiab]) OR cystatin C)) **AND** ("Metabolic Syndromes" or " Syndrome, Metabolic" or "Syndromes, Metabolic" or "Metabolic Syndrome X" or "Insulin Resistance Syndrome X" or "Syndrome X, Metabolic" or "Syndrome X, Insulin Resistance" or "Metabolic X Syndrome" or "Syndrome, Metabolic X" or "X Syndrome, Metabolic" or "Dysmetabolic Syndrome X " or"Reaven Syndrome X" or "Syndrome X, Reaven" or "Metabolic Cardiovascular Syndrome" or "Cardiovascular Syndrome, Metabolic" or "Syndrome, Metabolic Cardiovascular" or "metabolic Syndrome"[MeSH])

EMBASE
#1
'metabolic syndromes' OR 'syndrome, metabolic' OR 'syndromes, metabolic' OR 'metabolic syndrome x'/exp OR 'metabolic syndrome x' OR 'insulin resistance syndrome x' OR 'syndrome x, metabolic'/exp OR 'syndrome x, metabolic' OR 'syndrome x, insulin resistance' OR 'metabolic x syndrome' OR 'syndrome, metabolic x' OR 'x syndrome, metabolic' OR 'dysmetabolic syndrome x' OR 'syndrome x, dysmetabolic' OR 'reaven syndrome x' OR 'syndrome x, reaven' OR 'metabolic cardiovascular syndrome' OR 'cardiovascular syndrome, metabolic' OR 'cardiovascular syndromes, metabolic' OR 'syndrome, metabolic cardiovascular' OR 'metabolic syndrome'/exp OR 'metabolic syndrome'
#2
'kidney'/exp OR 'kidney function'/exp OR 'kidney function test'/exp OR 'kidney disease'/de OR 'chronic kidney disease'/exp OR 'chronic kidney failure'/exp OR 'cystatin c'/exp OR kidney*:ab,ti OR renal:ab,ti OR nephro*:ab,ti OR glomeru*:ab,ti OR ckd:ab,ti OR 'cystatin c':ab,ti
#3
#1 AND #2

Supplement S2. Conversion of HR or OR to RR

For studies with reported hazard ratios (HRs), values were treated as RRs.

For studies with low incidence of metabolic syndrome (MetS) (≤10%), or with reported odds ratios (ORs) between 0.5 and 2.5, values were treated as RRs.

For studies with reported ORs of less than 0.5 or greater than 2.5 with an incident of MetS greater than 10%, ORs were converted to RR using the following formulae and logic, as outlined by Zhang et al.[1]

$$OR = (\frac{P_1}{1 - P_1})/(\frac{P_0}{1 - P_0})$$

Thus,

$$\frac{P_1}{P_0} = \frac{OR}{[(1 - P_0) + (P_0 \times OR)]}$$

RR=$\frac{P_1}{P_0}$, therefore:

$$RR = \frac{OR}{[(1 - P_0) + (P_0 \times OR)]}$$

Notes: P_0 = incidence of the outcome of interest in the non-exposed group; P_1 = incidence of the outcome of interest in the exposed group. The lower and upper limits of the confidence interval were conversed also by applying this formula to the values of the confidence limits of OR.

Supplement S3. Characteristics of cohort studies that examined the relationship between MetS and kidney outcomes.

Study (author, year)	Country	Median follow up (years)	Mean age (years)	Male (%)	Participants	Definition of MetS	Kidney outcomes	Number of renal events	Adjusted covariates
Kurella *et al*, 2005[2]	US	9.0	53.7	44	10,096	NCEP ATP III	New-onset CKD defined as a GFR < 60 mL/min/1.73 m2	691	Age, sex, race, education, BMI, alcohol, tobacco use, coronary heart disease, and physical activity
Ninomiya *et al*, 2006[3]	Japan	5.0	57.7	41	1,440	Modified NCEP ATP III	New-onset CKD defined as a GFR < 60 mL/min/1.73 m2	88	Age, sex, baseline eGFR, proteinuria, serum albumin level, serum TC level, hemoglobin level, alcohol intake, smoking habits, and hyperinsulinemia.
Kitiyakara *et al*, 2007[4]	Thailand	12.0	42.4	76	2,067	NCEP ATP III	New-onset CKD defined as a GFR < 60 mL/min/1.73 m2	130	Age, sex, and smoking status
Rashidi *et al*, 2007[5]	Iran	3.0	39.3	42	4,607	NCEP ATP III	New-onset CKD defined as a GFR < 60 mL/min/1.73 m2	111	NA
Tozawa *et al*, 2007[6]	Japan	5.0	47.0	64	6,371	NCEP ATP III	New-onset CKD defined as dipstick-positive proteinuria (≥1+) or an eGFR <60 mL/min/1.73 m2	369	Age, sex, current cigarette smoking, and alcohol drinking habits
Lucove *et al*, 2008[7]	US	6.5	49.5	44	2,420	NCEP ATP III	New-onset CKD defined as the presence of ACR 46⩾30 mg/g or an eGFR ＜ 60 mL/min/1.73 m2	388	Age, sex, center, education, and smoking
Luk *et al*, 2008[8]	China	4.6	54.1	46	5,829	Modified NCEP ATP III	New-onset CKD defined as an eGFR < 60 ml/min per 1.73 m2 or the first hospitalization with CKD event	741	Age, sex, smoking status, duration of diabetes, HbA1C, BMI, and albuminuria
Ryu *et al*, 2009[9]	Korea	3.8	37.0	100	10,685	NCEP ATP III	New-onset CKD defined as a GFR < 60 mL/min/1.73 m2	291	Age, baseline eGRF, γ- glutamyltranspeptidase, uric acid levels, incidental hypertension, incidental diabetes, insulin

									resistance, hs-CRP, smoking, alcohol consumption and, regular exercise
Sun *et al*, 2010[10]	Chinese Taiwan	3.7	39.2	47	118,924	Modified NCEP ATP III, IDF	New-onset CKD defined as dipstick-positive proteinuria (≥1+) or an eGFR <60 mL/min/1.73 m2	12,672	Age, sex, check-up centers and, current smoking
Watanabe *et al*, 2010[11]	Japan	5.8	59.0	34	34,986	Modified NCEP ATP III	New-onset CKD defined as a GFR < 60 mL/min/1.73 m2	930	Age and sex
Cheng *et al*,2012[12]	Chinese Taiwan	3.2	73.1	57	1,456	NCEP ATP III	Rapid decline in kidney function as an annual decline in GFR greater than 3 ml/min/1.73 m2	NA	Age, sex, hemoglobin, serum albumin, globulin and, uric acid
							New-onset CKD defined as dipstick-positive proteinuria (≥1+) or an eGFR <60 mL/min/1.73 m2	67	
Navaneethan *et al*, 2013[13]	US	2.2	72.3	46	25,868	NHLBI/AHA and IDF	ESRD defined using hospital records	894	Age, sex, race, smoking, malignancy, congestive heart failure, cerebrovascular disease, CVD, chronic obstructive pulmonary disease, use of ACEI/ARB, LDL-C, hemoglobin, albumin and baseline eGFR
Li *et al*, 2015[14]	China	7.1	50.4	53	2,696	NHLBI/AHA and IDF	Rapid decline in kidney function as an annual decline in GFR greater than 3 ml/min/1.73 m2	533	Age, sex, baseline eGFR, current smoking, current drinking, TC, and physical activity
							New-onset CKD defined as an	47	

							eGFR <60 mL/min/1.73 m2		
Song *et al*, 2015[15]	Korea	3.7	48.7	38	1,881	NHLBI/AHA	New-onset CKD defined as an eGFR <60 mL/min/1.73 m2	62	Sex, alcohol use, smoking amount, physical activity and BMI
Nishikawa *et al*, 2015[16]	Japan	7.8	46.9	86	23,894	Modified NCEP ATP III	New-onset CKD defined as an eGFR <60 mL/min/1.73 m2	1764	Age, sex, smoking status, alcohol consumption, exercise habits, walking time in commutation, type of work and occupational exposure.
Huh *et al*, 2017[17]	Korea	10.0	51.7	47	6,065	Modified NCEP-ATP III	Rapid decline in kidney function as an annual decline in GFR greater than 3 ml/min/1.73 m2	1508	Age, sex, baseline eGFR, smoking status, physical activity, alcohol, protein intake, TC and, hs-CRP
							New-onset CKD defined as an eGFR <60 mL/min/1.73 m2	893	
Stefansson *et al*, 2018[18]	Norway	5.6	58.0	49	1,261	NHLBI/AHA and IDF	Rapid decline in kidney function as an annual decline in GFR greater than 3 ml/min/1.73 m2	129	Age, sex, baseline smoking, LDL-C, cholesterol-lowering medication use, resting heart rate, nonsteroid anti-inflammatory drug use, and baseline urinary albumin-creatinine ratio.
Hayashi *et al*, 2017[19]	Japan	2.0	58.9	64	2,531	the Joint Interim Statement 2009 definition	eGFR decline defined as eGFR reduction rate of 15% or more over 1 year and/or new-onset eGFR ＜60 ml/min/m2	NA	Age, sex, baseline eGFR and smoking
Yun *et al*, 2018[20]	Korea	3.1	53.5	61	1,940	metabolic abnormality	A composite of a 50% decline in eGRF from the baseline value or ESRD	395	Age, sex, smoking status, hemoglobin, albumin, parathyroid hormone, and adiponectin levels, left ventricular mass index, eGFR and proteinuria
Ding *et al*, 2018[21]	China	3.0	43.9	46.6	63,680	Modified NCEP ATP III	New-onset CKD defined as dipstick-positive proteinuria (≥1+) or an eGFR <60	6,714	Age, sex, education, smoking status, alcohol consumption, physical activity, hypertension family history, and family history of diabetes

							mL/min/1.73 m2		
Kawamoto *et al*, 2019[22]	Japan	3.0	67.9	43	959	Modified NCEP-ATP III	Rapid decline in kidney function as an annual decline in GFR greater than 1.2 ml/min/1.73 m2	54	Age, sex, prevalence of obesity, smoking status, alcohol consumption, prevalence of CVD, LDL-C, serum uric acid, and eGFR
							New-onset CKD defined as an eGFR <60 mL/min/1.73 m2	130	
Hu *et al*, 2019[23]	China	3.0	57.8	26	7,231	Modified NCEP-ATP III	Rapid decline in kidney function as (final eGFR–initial eGFR)/ (initial eGFR) × 100% ≤30%	886	Age, sex, smoking, tea consumption, drinking, hypertension, and diabetes-related drugs
							New-onset CKD defined as an eGFR <60 mL/min/1.73 m2	172	
Chuang *et al*, 2019[24]	China	5.0	64.6	46	935	Modified NCEP-ATP III	Worsening renal function defined as the doubling of serum creatinine or eGFR reduction more than 50% from baseline	41	Age, gender, diabetes duration, HbA1c level, TC, LDL-C, BMI, hypertension, use of ACEI or ARB, CKD and microalbuminuria
Wang *et al*, 2020[25]	China	4.6	62.8	45	15,229	NECP ATP III	New-onset CKD defined as an eGFR <60 mL/min/1.73 m2	1151	Age, sex, race, marital status, education level, lifestyle factors (smoking, passive smoking, drinking, and physical activity), baseline BMI, history of CVD, uric acid, alanineaminotransferase and baseline eGFR.
Shih *et al*, 2020[26]	China	5.0	72.0	43	460	Modified NCEP ATP III	Worsening renal function defined as a 50% reduction in eGFR, doubling of serum creatinine or ESRD	23	Diabetes duration and HbA1c
Barbieri *et al*,	Spain	7.4	NA	61	166	with 3 metabolic	Renal event defined as doubling	39	Age, sex, obesity, eGFR, albuminuria, diabetes, systolic

2021[27]						factors	serum creatinine, an eGFR decrease ≥50% or initiation of renal replacement therapy		blood pressure, use of ACEI/ARB, statin and aspirin treatment.
Wu *et al*, 2021[28]	China	4.0	58.9	45	5,752	Chinese guidelines for the management of dyslipidemia in adults	New-onset CKD defined as an eGFR <60 mL/min/1.73 m2	163	Age, sex, residence area, education level, BMI, smoking status, alcohol consumption, systolic blood pressure, triglycerides, fasting glucose, waist, and heart diseases
Wu *et al*, 2019[29]	China	2.3	55.8	35	3,108	NHLBI/AHA and IDF	Rapid decline in kidney function as an annual decline in GFR greater than 3 ml/min/1.73 m2	225	Age, sex, eGFR, smoking, drinking, and LDL-C
Zhao *et al*, 2022[30]	China	2.3	50.0	70	411	Modified NCEP ATP III	ESRD defined as eGFR < 15 mL/min/1.73 m2 or the need for chronic renal replacement therapy	163	Age, sex, baseline eGFR, 24-h proteinuria, uric acid, HbA1c, serum albumin concentration and pathological parameters
Cao *et al*, 2015[31]	China	4.5	54.1	54	6,852	NECP ATP III	New-onset CKD defined as dipstick-positive proteinuria (≥1+) or an eGFR <60 mL/min/1.73 m2	776	Age, sex, smoking, plasma LDL-C level, medication use, and physical inactivity
Deboer *et al*, 2018[32]	US	8.0	52.8	37	2,627	Modified NCEP ATP III	New-onset CKD defined as an eGFR <60 mL/min/1.73 m2	207	Age, sex, physical activity, nutrition, and current smoking status
							Rapid decline in kidney function as (final eGFR–initial eGFR)/ (initial eGFR) × 100% ≤16.8%	685	
Chang et al,	Korea	3.2	60.5	61	41,194	NECP ATP III	New-onset CKD defined as an	356	Age, sex, baseline eGFR, history of CVD, drinking,

2015[33]	eGFR <60 mL/min/1.73 m2	smoking, exercise habits, alanineaminotransferase, γ-glutamyltranspeptidase, LDL-C, uric acid, and hs-CRP.

ACEI/ARB, angiotensin converting enzyme inhibitor or angiotensin receptor blocker; BMI, body mass index; CKD, chronic kidney disease; CVD, cardiovascular disease; eGFR, estimated glomerular filtration rate; HbA1C, hemoglobin A1c; hs-CRP, high-sensitivity C-reactive protein; LDL-C, low-density lipoprotein cholesterol; NHLBI/AHA, National Heart, Lung and Blood Institute/American Heart Association; NCEP ATP III, National Cholesterol Education Program Adult Treatment Panel III; MetS, metabolic syndrome; TC, total cholesterol.

Supplement S4. Newcastle-Ottawa quality assessments for studies included in the meta-analysis.

	Selection				Comparability	Outcome			
**Study (first author, year)**	Representativeness of the exposed cohort	Selection of the non-exposed cohort	Ascertainment of exposure	Demonstration that outcome of interest was not present at start of study	Comparability of cohorts on the basis of the design or analysis	Assessment of outcome	Was follow-up long enough for outcomes to occur	Adequacy of follow up of cohorts	Total
Kurella *et al*, 2005	1	1	1	1	2	1	1	1	9
Ninomiya *et al*, 2006	1	1	1	1	2	1	1	1	9
Kitiyakara *et al*, 2007	1	1	1	1	2	1	1	1	9
Rashidi *et al*, 2007	1	1	1	1	2	1	0	0	7
Tozawa *et al*, 2007	1	1	1	1	2	1	1	0	8
Lucove *et al*, 2008	1	1	1	1	2	1	1	1	9
Luk *et al*, 2008	1	1	1	1	2	1	0	0	7
Ryu *et al*, 2009	1	1	1	1	2	1	0	0	7
Sun *et al*, 2010	1	1	1	1	2	1	1	0	8
Watanabe *et al*, 2010	1	1	1	1	2	1	1	0	8
Cheng *et al*,2012	1	1	1	1	2	1	0	0	7
Navaneethan *et al*, 2013	1	1	1	1	2	1	1	1	9
Li *et al*, 2015	1	1	1	1	2	1	1	0	8
Song *et al*, 2015	0	1	1	1	2	1	1	0	7
Nishikawa *et al*, 2015	1	1	1	1	2	1	1	0	8
Huh *et al*, 2017	1	1	1	1	2	1	1	1	9
Stefansson *et al*, 201	1	1	1	1	2	1	1	1	9
Hayashi *et al*, 2017	1	1	1	1	2	1	0	0	7
Yun *et al*, 2018	1	1	1	1	2	1	1	1	9

Ding *et al*, 2018	1	1	1	1	2	1	0	0	7
Kawamoto *et al*, 2019	1	1	1	1	2	1	0	0	7
Hu *et al*, 2019	1	1	1	1	2	1	1	1	9
Chuang *et al*, 2019	1	1	1	1	2	1	1	0	8
Wang *et al*, 2020	1	1	1	1	2	1	1	0	8
Shih *et al*, 2020	1	1	1	1	2	1	1	0	8
Barbieri *et al*, 2021	0	1	1	1	2	1	1	0	7
Wu *et al*, 2021	1	1	1	1	2	1	1	1	9
Wu *et al*, 2019	1	1	1	1	2	1	0	0	7
Zhao *et al*, 2022	1	1	1	1	2	1	0	0	7
Cao *et al*, 2015	1	1	1	1	1	1	1	0	7
Deboer *et al*, 2018	1	1	1	1	1	1	1	0	7
Chang et al, 2015	1	1	1	1	2	1	0	0	7

Supplement S5. Sensitivity analysis for the association between metabolic syndrome and renal function.

Study name	Statistics with study removed			
	Point	Lower limit	Upper limit	p-Value
Kurella 2005	1.50	1.39	1.62	0.000
Ninomiya 2006	1.49	1.38	1.60	0.000
Tozawa 2007	1.48	1.38	1.60	0.000
Rashidi 2007	1.49	1.38	1.60	0.000
Kitiyakara 2007	1.50	1.39	1.61	0.000
Luk 2008	1.51	1.40	1.63	0.000
Lucove 2008	1.51	1.40	1.62	0.000
Watanabe 2010	1.46	1.36	1.56	0.000
Ryu 2009	1.48	1.38	1.60	0.000
Sun 2010	1.52	1.40	1.65	0.000
Navaneethan 2013	1.50	1.39	1.62	0.000
Song 2015	1.49	1.39	1.61	0.000
Nishikawa 2015	1.49	1.38	1.61	0.000
Li 2015	1.49	1.38	1.60	0.000
Cao 2015	1.47	1.37	1.58	0.000
Chang 2015	1.51	1.40	1.62	0.000
Huh 2017	1.50	1.39	1.62	0.000
Hayashi 2017	1.50	1.39	1.62	0.000
Ding 2018	1.52	1.40	1.64	0.000
DeBoer 2018	1.52	1.41	1.63	0.000
Stefansson 2018	1.50	1.39	1.62	0.000
Yun 2018	1.50	1.39	1.62	0.000
Hu 2019	1.49	1.38	1.60	0.000
Chuang 2019	1.49	1.39	1.60	0.000
Wang 2020	1.50	1.39	1.62	0.000
Shih 2020	1.49	1.39	1.60	0.000
Barbieri 2021	1.50	1.40	1.62	0.000
Zhao 2022	1.49	1.38	1.60	0.000
Kawamoto 2019	1.48	1.38	1.59	0.000
Wu 2019	1.49	1.38	1.60	0.000
Cheng 2012	1.51	1.41	1.63	0.000
Wu 2021	1.49	1.39	1.61	0.000
	1.50	1.39	1.61	0.000

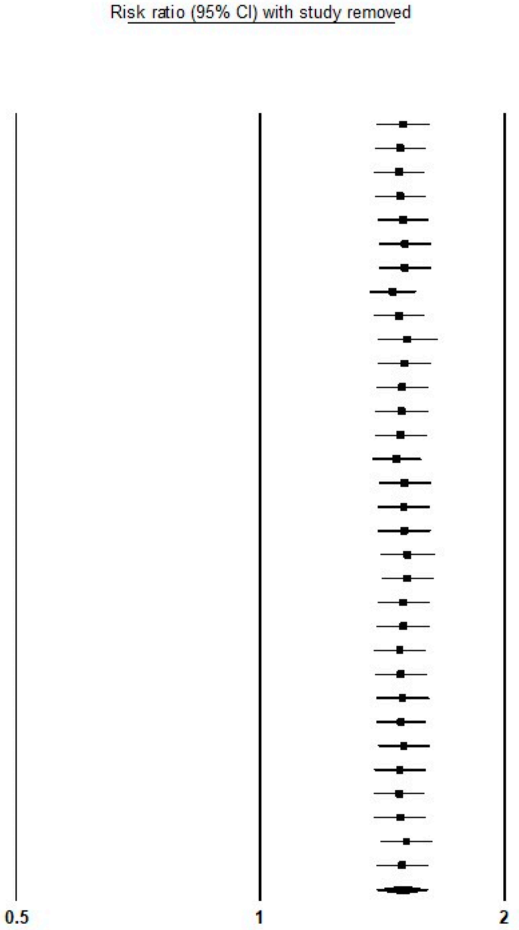

Supplement S6. Sensitivity analysis for the association between metabolic syndrome components and renal dysfunction.

Risk factor	Subgroup	Number of studies/participants	Test of association	Test of heterogeneity	
			OR (95%CI)	I^2(%)	P
IFG	Overall	18/362,546	1.20 (1.09–1.33)	85.82	<0.001
	Age range				
	≥40 years	7/48,937	1.29 (1.02-1.63)	84.46	<0.001
	Median follow-up				0.204
	<5 years	12/302,332	1.15 (1.34-1.28)	83.94	
	≥5 years	6/60,214	1.32 (1.11-1.56)	76.29	
	Country				0.992
	Asian	15/321,975	1.23 (1.06-1.43)	85.01	
	Non-Asian	3/40,571	1.23 (0.92-1.63)	92.17	
	Diabetic status				0.059
	Diabetics	1/7231	1.39 (1.00-1.93)	0.00	
	Non-diabetics	4/214,363	1.01 (0.94-1.08)	64.93	
	Baseline renal function				0.007
	Normal	14/329,418	1.12 (1.01-1.24)	81.27	
	Reduced	4/33,128	1.47 (1.22-1.75)	78.07	
Elevated BP	Overall	20/362,875	1.42 (1.27-1.59)	87.42	<0.001
	Age range				
	≥40 years	8/49,397	1.28 (1.19-1.72)	75.97	<0.001
	Median follow-up				<0.001
	<5 years	15/308,572	1.35 (1.20-1.52)	82.12	
	≥5 years	5/54,303	1.50 (1.33-1.93)	85.86	
	Country				0.001
	Asian	17/322,304	1.33 (1.20-1.48)	83.50	
	Non-Asian	3/40,571	2.25 (1.70-2.98)	63.33	
	Diabetic status				0.733
	Diabetics	5/207,992	1.43 (1.16-1.76)	95.11	
	Non-diabetics	4/13,931	1.35 (1.01-1.79)	0.00	

	Baseline renal function				0.909
	Normal	14/328,876	1.43 (1.26-1.62)	90.65	
	Reduced	6/33,999	1.41 (1.11-1.78)	48.68	
Obesity	Overall	19/339,572	1.27 (1.19-1.37)	68.78	<0.001
	Age range				
	≥40 years	7/116,820	1.35 (1.12-1.62)	75.82	<0.001
	Median follow-up				0.420
	<5 years	13/278,097	1.30 (1.19-1.43)	76.08	
	≥5 years	6/61,475	1.22 (1.07-1.39)	33.03	
	Country				0.176
	Asian	17/328,215	1.30 (1.20-1.40)	70.71	
	Non-Asian	2/11,357	1.11 (0.90-1.37)	0.00	
	Diabetic status				0.219
	Diabetics	2/7642	1.39 (1.07-1.82)	15.83	
	Non-diabetics	6/211,017	1.17 (1.07-1.28)	68.95	
	Baseline renal function				0.813
	Normal	15/116,820	1.27 (1.17-1.37)	74.53	
	Reduced	4/7671	1.30 (1.18-1.37)	0.00	
Increased triglycerides	Overall	20/368,786	1.25 (1.15–1.36)	92.93	<0.001
	Age range				
	≥40 years	7/48,937	1.33 (1.23-1.44)	0.00	0.690
	Median follow-up				0.077
	<5 years	15/308,572	1.20 (1.10-1.31)	92.30	
	≥5 years	5/60,214	1.41 (1.21-1.65)	65.04	
	Country				0.007
	Asian	17/328,215	1.28 (1.20-1.37)	55.62	
	Non-Asian	3/40,571	1.09 (0.98-1.20)	86.03	
	Diabetic status				0.328
	Diabetics	3/13,471	1.35 (1.09-1.67)	31.70	

	Non-diabetics	6/214,363	1.19 (1.05-1.35)	96.64	
	Baseline renal function				0.349
	Normal	15/335,247	1.28 (1.16-1.40)	94.35	
	Reduced	5/33,539	1.15 (0.94-1.40)	0.00	
Low HDL-C	Overall	18/337,089	1.24 (1.15–1.34)	87.19	<0.001
	Age range				
	≥40 years	6/102,006	1.24 (1.08-1.42)	54.12	0.053
	Median follow-up				0.103
	＜5 years	13/276,875	1.18 (1.09-1.28)	85.61	
	≥5 years	5/60,214	1.34 (1.18-1.51)	63.33	
	Country				0.215
	Asian	15/319,966	1.29 (1.17-1.42)	82.29	
	Non-Asian	3/17,123	1.11 (0.90-1.38)	87.84	
	Diabetic status				0.027
	Diabetics	2/7642	1.59 (1.20-2.10)	0.00	
	Non-diabetics	6/211,017	1.14 (1.05-1.24)	91.29	
	Baseline renal function				0.330
	Normal	14/329,418	1.26 (1.16-1.37)	89.92	
	Reduced	4/7671	1.31 (0.93-1.38)	41.48	

Renal dysfunction is defined as a composite of rapid eGFR decline, new-onset CKD, 50% reduction in eGFR or doubling of serum creatinine and ESRD. BP, blood pressure; HDL-C, high density lipoprotein cholesterol; IFG, impaired fasting glucose.

Supplement S7. Funnel plot showing publication bias of studies on the association between metabolic syndrome and renal dysfunction.

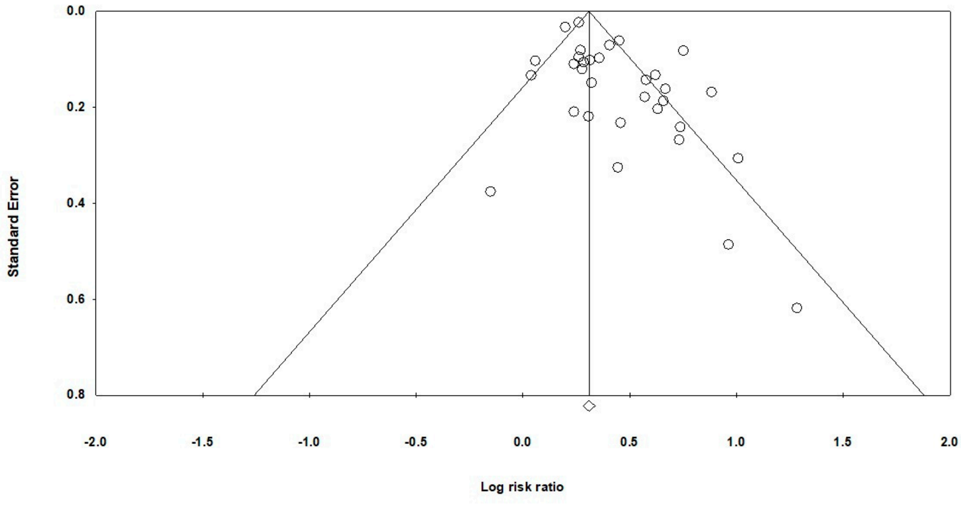

Reference

1. Zhang, J.; Yu, K.F. What's the relative risk? A method of correcting the odds ratio in cohort studies of common outcomes. *Jama* **1998**, *280*, 1690-1691, doi:10.1001/jama.280.19.1690.
2. Kurella, M.; Lo, J.C.; Chertow, G.M. Metabolic syndrome and the risk for chronic kidney disease among nondiabetic adults. *J Am Soc Nephrol* **2005**, *16*, 2134-2140, doi:10.1681/asn.2005010106.
3. Ninomiya, T.; Kiyohara, Y.; Kubo, M.; Yonemoto, K.; Tanizaki, Y.; Doi, Y.; Hirakata, H.; Iida, M. Metabolic syndrome and CKD in a general Japanese population: the Hisayama Study. *Am J Kidney Dis* **2006**, *48*, 383-391, doi:10.1053/j.ajkd.2006.06.003.
4. Kitiyakara, C.; Yamwong, S.; Cheepudomwit, S.; Domrongkitchaiporn, S.; Unkurapinun, N.; Pakpeankitvatana, V.; Sritara, P. The metabolic syndrome and chronic kidney disease in a Southeast Asian cohort. *Kidney Int* **2007**, *71*, 693-700, doi:10.1038/sj.ki.5002128.
5. Rashidi, A.; Ghanbarian, A.; Azizi, F. Are patients who have metabolic syndrome without diabetes at risk for developing chronic kidney disease? Evidence based on data from a large cohort screening population. *Clin J Am Soc Nephrol* **2007**, *2*, 976-983, doi:10.2215/cjn.01020207.
6. Tozawa, M.; Iseki, C.; Tokashiki, K.; Chinen, S.; Kohagura, K.; Kinjo, K.; Takishita, S.; Iseki, K. Metabolic syndrome and risk of developing chronic kidney disease in Japanese adults. *Hypertens Res* **2007**, *30*, 937-943, doi:10.1291/hypres.30.937.
7. Lucove, J.; Vupputuri, S.; Heiss, G.; North, K.; Russell, M. Metabolic syndrome and the development of CKD in American Indians: the Strong Heart Study. *Am J Kidney Dis* **2008**, *51*, 21-28, doi:10.1053/j.ajkd.2007.09.014.
8. Luk, A.O.; So, W.Y.; Ma, R.C.; Kong, A.P.; Ozaki, R.; Ng, V.S.; Yu, L.W.; Lau, W.W.; Yang, X.; Chow, F.C.; et al. Metabolic syndrome predicts new onset of chronic kidney disease in 5,829 patients with type 2 diabetes: a 5-year prospective analysis of the Hong Kong Diabetes Registry. *Diabetes Care* **2008**, *31*, 2357-2361, doi:10.2337/dc08-0971.
9. Ryu, S.; Chang, Y.; Woo, H.Y.; Lee, K.B.; Kim, S.G.; Kim, D.I.; Kim, W.S.; Suh, B.S.; Jeong, C.; Yoon, K. Time-dependent association between metabolic syndrome and risk of CKD in Korean men without hypertension or diabetes. *Am J Kidney Dis* **2009**, *53*, 59-69, doi:10.1053/j.ajkd.2008.07.027.
10. Sun, F.; Tao, Q.; Zhan, S. Metabolic syndrome and the development of chronic kidney disease among 118 924 non-diabetic Taiwanese in a retrospective cohort. *Nephrology (Carlton)* **2010**, *15*, 84-92, doi:10.1111/j.1440-1797.2009.01150.x.
11. Watanabe, H.; Obata, H.; Watanabe, T.; Sasaki, S.; Nagai, K.; Aizawa, Y. Metabolic syndrome and risk of development of chronic kidney disease: the Niigata preventive medicine study. *Diabetes Metab Res Rev* **2010**, *26*, 26-32, doi:10.1002/dmrr.1058.
12. Cheng, H.T.; Huang, J.W.; Chiang, C.K.; Yen, C.J.; Hung, K.Y.; Wu, K.D. Metabolic syndrome and insulin resistance as risk factors for development of chronic kidney disease and rapid decline in renal function in elderly. *J Clin Endocrinol Metab* **2012**, *97*, 1268-1276, doi:10.1210/jc.2011-2658.
13. Navaneethan, S.D.; Schold, J.D.; Kirwan, J.P.; Arrigain, S.; Jolly, S.E.; Poggio, E.D.; Beddhu,

S.; Nally, J.V., Jr. Metabolic syndrome, ESRD, and death in CKD. *Clin J Am Soc Nephrol* **2013**, *8*, 945-952, doi:10.2215/cjn.09870912.
14. Li, Y.; Xie, D.; Qin, X.; Tang, G.; Xing, H.; Li, Z.; Xu, X.; Xu, X.; Hou, F. Metabolic syndrome, but not insulin resistance, is associated with an increased risk of renal function decline. *Clin Nutr* **2015**, *34*, 269-275, doi:10.1016/j.clnu.2014.04.002.
15. Song, Y.M.; Sung, J.; Lee, K. Longitudinal relationships of metabolic syndrome and obesity with kidney function: Healthy Twin Study. *Clin Exp Nephrol* **2015**, *19*, 887-894, doi:10.1007/s10157-015-1083-5.
16. Nishikawa, K.; Takahashi, K.; Okutani, T.; Yamada, R.; Kinaga, T.; Matsumoto, M.; Yamamoto, M. Risk of chronic kidney disease in non-obese individuals with clustering of metabolic factors: a longitudinal study. *Intern Med* **2015**, *54*, 375-382, doi:10.2169/internalmedicine.54.3092.
17. Huh, J.H.; Yadav, D.; Kim, J.S.; Son, J.W.; Choi, E.; Kim, S.H.; Shin, C.; Sung, K.C.; Kim, J.Y. An association of metabolic syndrome and chronic kidney disease from a 10-year prospective cohort study. *Metabolism* **2017**, *67*, 54-61, doi:10.1016/j.metabol.2016.11.003.
18. Stefansson, V.T.N.; Schei, J.; Solbu, M.D.; Jenssen, T.G.; Melsom, T.; Eriksen, B.O. Metabolic syndrome but not obesity measures are risk factors for accelerated age-related glomerular filtration rate decline in the general population. *Kidney Int* **2018**, *93*, 1183-1190, doi:10.1016/j.kint.2017.11.012.
19. Hayashi, K.; Takayama, M.; Abe, T.; Kanda, T.; Hirose, H.; Shimizu-Hirota, R.; Shiomi, E.; Iwao, Y.; Itoh, H. Investigation of Metabolic Factors Associated with eGFR Decline Over 1 Year in a Japanese Population without CKD. *J Atheroscler Thromb* **2017**, *24*, 863-875, doi:10.5551/jat.38612.
20. Yun, H.R.; Kim, H.; Park, J.T.; Chang, T.I.; Yoo, T.H.; Kang, S.W.; Choi, K.H.; Sung, S.; Kim, S.W.; Lee, J.; et al. Obesity, Metabolic Abnormality, and Progression of CKD. *Am J Kidney Dis* **2018**, *72*, 400-410, doi:10.1053/j.ajkd.2018.02.362.
21. Ding, C.; Yang, Z.; Wang, S.; Sun, F.; Zhan, S. The associations of metabolic syndrome with incident hypertension, type 2 diabetes mellitus and chronic kidney disease: a cohort study. *Endocrine* **2018**, *60*, 282-291, doi:10.1007/s12020-018-1552-1.
22. Kawamoto, R.; Akase, T.; Ninomiya, D.; Kumagi, T.; Kikuchi, A. Metabolic syndrome is a predictor of decreased renal function among community-dwelling middle-aged and elderly Japanese. *Int Urol Nephrol* **2019**, *51*, 2285-2294, doi:10.1007/s11255-019-02320-0.
23. Hu, Y.; Shi, L.X.; Zhang, Q.; Peng, N.C. Increased Risk of Chronic Kidney Diseases in Patients with Metabolic Syndrome: A 3-year Prospective Cohort Study. *Curr Med Sci* **2019**, *39*, 204-210, doi:10.1007/s11596-019-2020-8.
24. Chuang, S.M.; Shih, H.M.; Chien, M.N.; Liu, S.C.; Wang, C.H.; Lee, C.C. Risk factors in metabolic syndrome predict the progression of diabetic nephropathy in patients with type 2 diabetes. *Diabetes Res Clin Pract* **2019**, *153*, 6-13, doi:10.1016/j.diabres.2019.04.022.
25. Wang, Y.; Sun, B.; Sheng, L.T.; Pan, X.F.; Zhou, Y.; Zhu, J.; Li, X.; Yang, K.; Guo, K.; Zhang, X.; et al. Association between weight status, metabolic syndrome, and chronic kidney disease among middle-aged and elderly Chinese. *Nutr Metab Cardiovasc Dis* **2020**, *30*, 2017-2026, doi:10.1016/j.numecd.2020.06.025.
26. Shih, H.M.; Chuang, S.M.; Lee, C.C.; Liu, S.C.; Tsai, M.C. Addition of Metabolic Syndrome

to Albuminuria Provides a New Risk Stratification Model for Diabetic Kidney Disease Progression in Elderly Patients. *Sci Rep* **2020**, *10*, 6788, doi:10.1038/s41598-020-63967-9.

27. Barbieri, D.; Goicoechea, M.; García-Prieto, A.; Delgado, A.; Verde, E.; Verdalles, U.; Pérez de José, A.; Carbayo, J.; Muñoz de Morales, A.; Luño, J. Obesity related risk for chronic kidney disease progression and cardiovascular disease after propensity score matching. *Hipertens Riesgo Vasc* **2021**, *38*, 63-71, doi:10.1016/j.hipert.2020.09.004.
28. Wu, N.; Qin, Y.; Chen, S.; Yu, C.; Xu, Y.; Zhao, J.; Yang, X.; Li, N.; Pan, X.F. Association between metabolic syndrome and incident chronic kidney disease among Chinese: A nation-wide cohort study and updated meta-analysis. *Diabetes Metab Res Rev* **2021**, *37*, e3437, doi:10.1002/dmrr.3437.
29. Wu, Z.; Jiang, Y.; Jia, J.; He, D.; Sun, P.; Li, J.; Huo, Y.; Fan, F.; Zhang, Y. Metabolic Syndrome Is Associated With Rapid Estimated Glomerular Filtration Rate Decline In A Chinese Community-Based Population. *Diabetes Metab Syndr Obes* **2019**, *12*, 2085-2093, doi:10.2147/dmso.S217326.
30. Zhao, L.; Zou, Y.; Bai, L.; Zhou, L.; Ren, H.; Wu, Y.; Wang, Y.; Li, S.; Su, Q.; Tang, L.; et al. Prognostic value of metabolic syndrome in renal structural changes in type 2 diabetes. *Int Urol Nephrol* **2022**, *54*, 2005-2014, doi:10.1007/s11255-021-03051-x.
31. Cao, X.; Zhou, J.; Yuan, H.; Wu, L.; Chen, Z. Chronic kidney disease among overweight and obesity with and without metabolic syndrome in an urban Chinese cohort. *BMC Nephrol* **2015**, *16*, 85, doi:10.1186/s12882-015-0083-8.
32. DeBoer, M.D.; Filipp, S.L.; Musani, S.K.; Sims, M.; Okusa, M.D.; Gurka, M. Metabolic Syndrome Severity and Risk of CKD and Worsened GFR: The Jackson Heart Study. *Kidney Blood Press Res* **2018**, *43*, 555-567, doi:10.1159/000488829.
33. Jung, C.H.; Lee, M.J.; Kang, Y.M.; Hwang, J.Y.; Kim, E.H.; Park, J.Y.; Kim, H.K.; Lee, W.J. The risk of chronic kidney disease in a metabolically healthy obese population. *Kidney Int* **2015**, *88*, 843-850, doi:10.1038/ki.2015.183.
